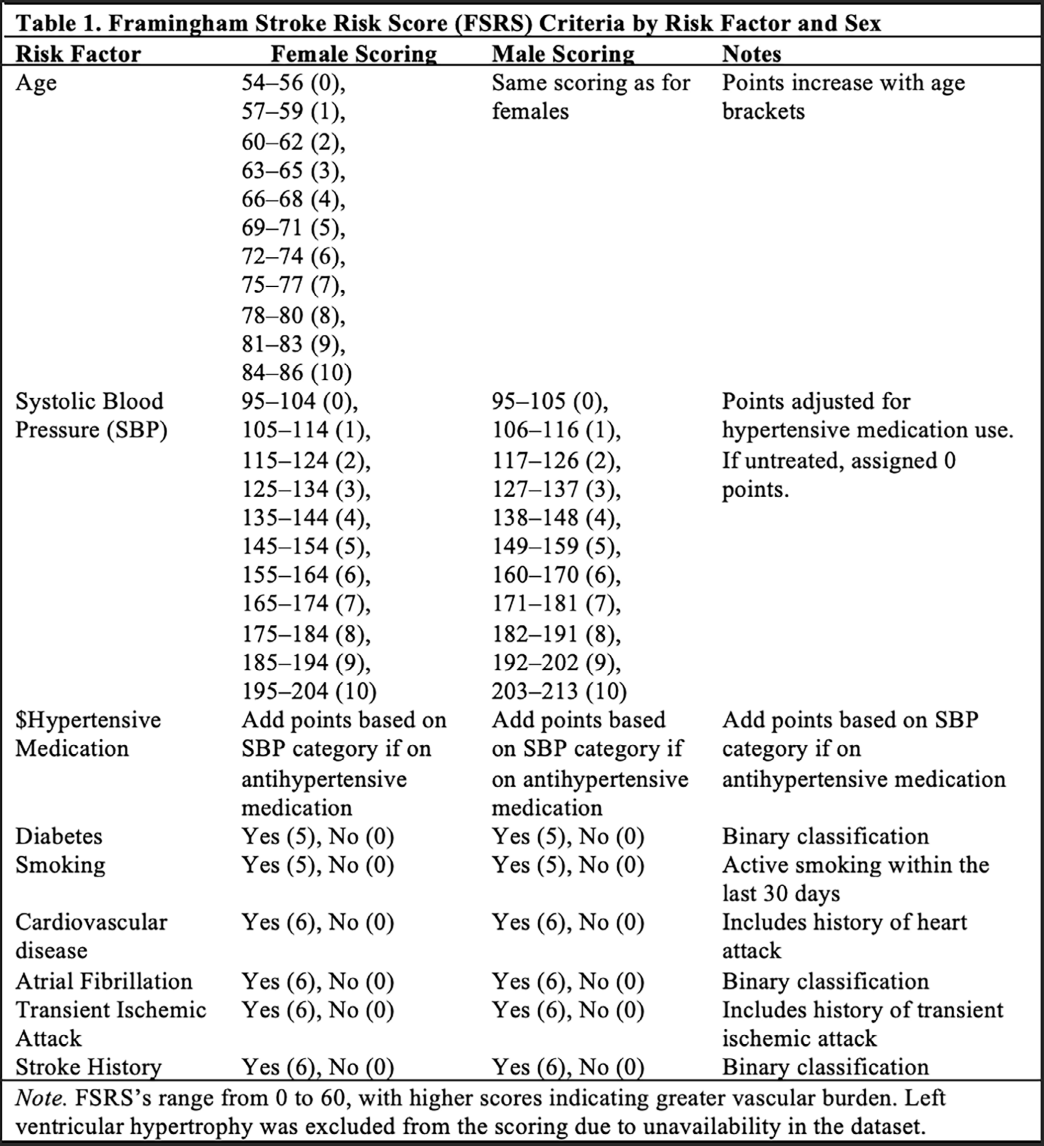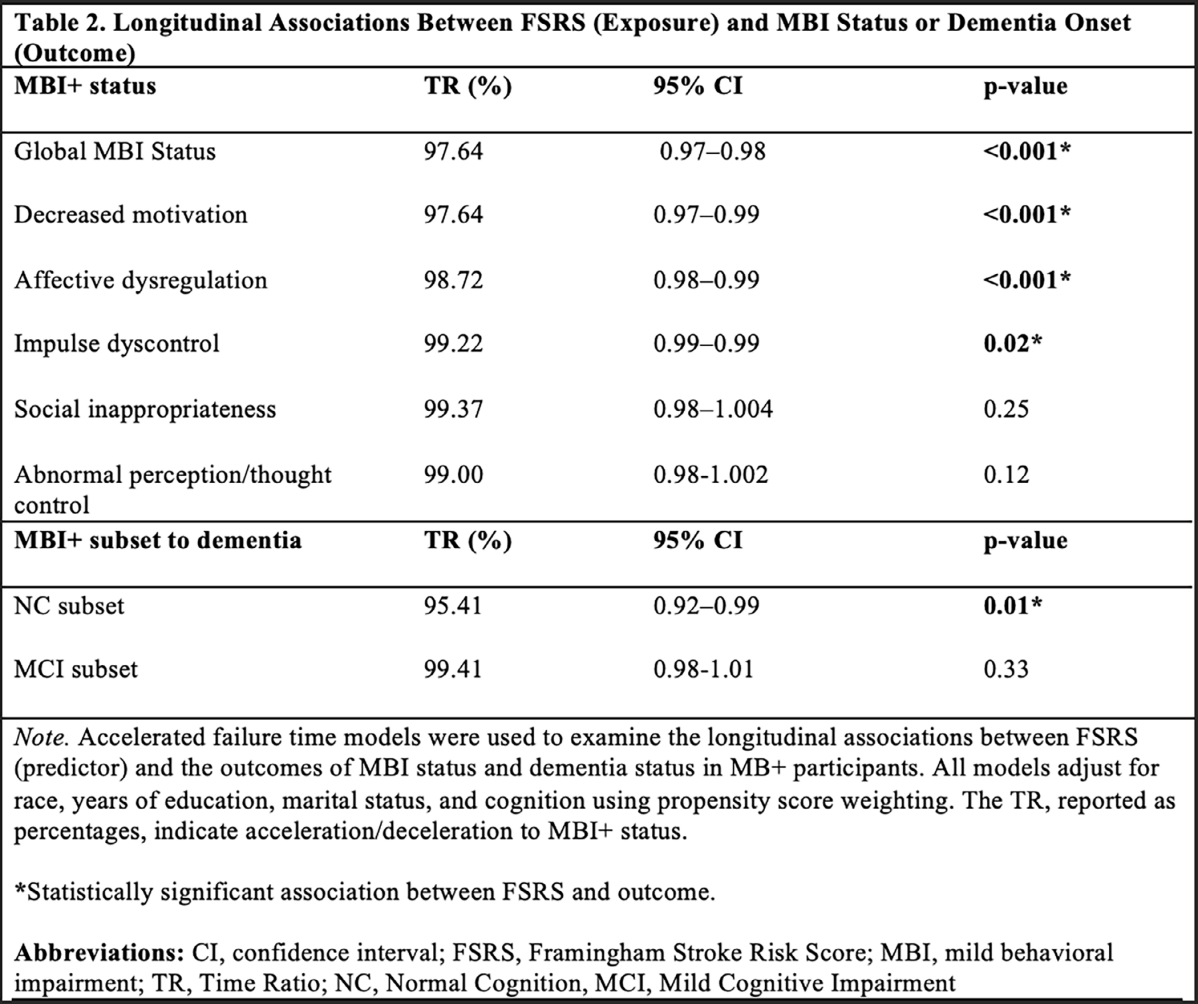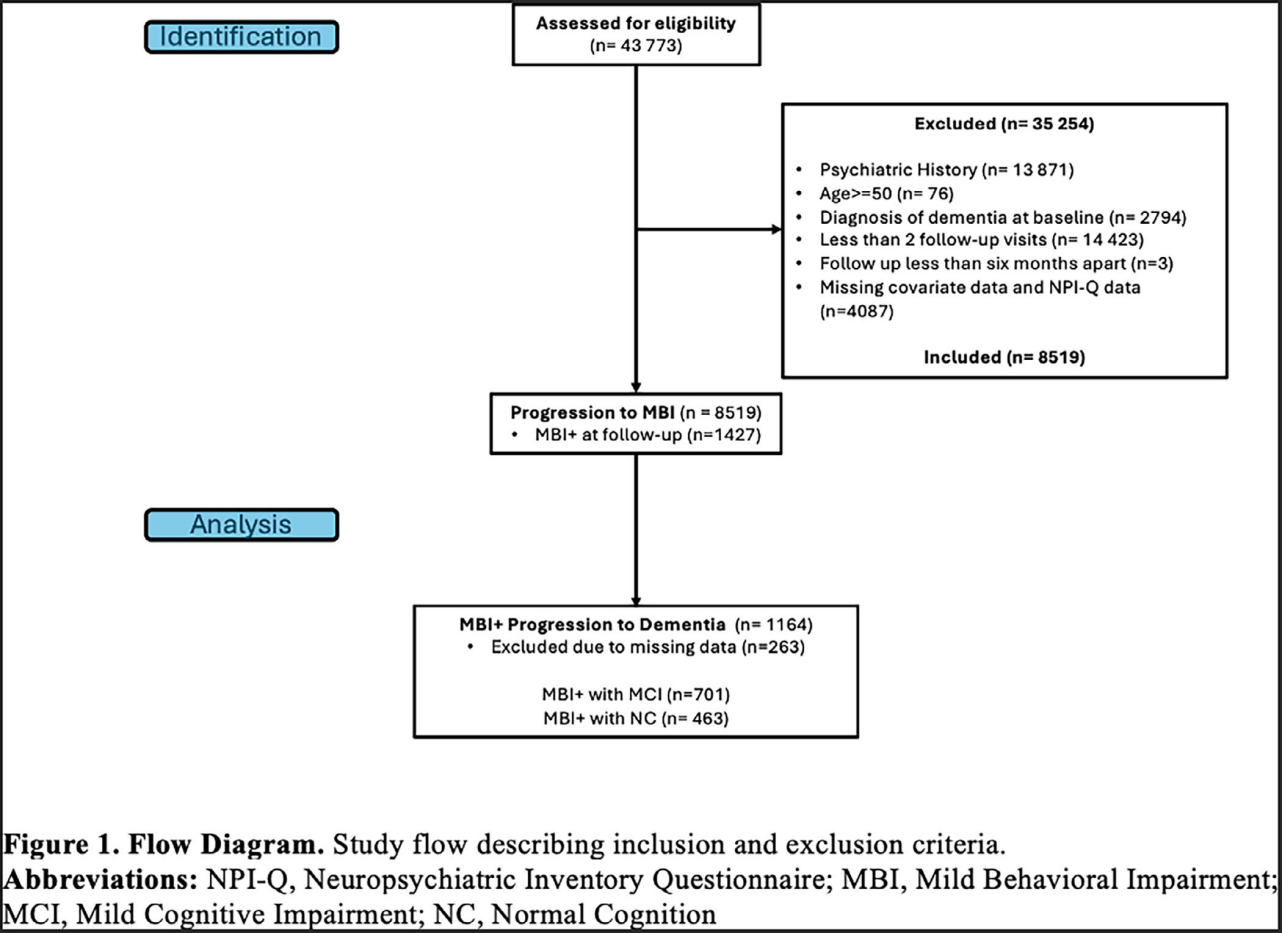# Framingham Stroke Risk Score, Mild Behavioral Impairment, and Progression to Dementia

**DOI:** 10.1002/alz70860_102161

**Published:** 2025-12-23

**Authors:** Ibadat Warring, Dylan X. Guan, Maryam Ghahremani, Eric E. Smith, Zahinoor Ismail

**Affiliations:** ^1^ University of Calgary, Calgary, AB, Canada; ^2^ Hotchkiss Brain Institute, University of Calgary, Calgary, AB, Canada

## Abstract

**Background:**

Vascular risk factors (VRFs) contribute to dementia risk through cerebrovascular damage, potentially accelerating dementia onset. Mild behavioral impairment (MBI) identifies dementia risk by leveraging later‐life emergent and persistent neuropsychiatric symptoms. This study examined the relationship between vascular burden and progression to MBI and dementia, in MBI‐free dementia‐free older adults.

**Method:**

Data from 8519 dementia‐free participants aged ≥50 years were acquired from the National Alzheimer's Coordinating Center (Figure 1). A composite measure of VRFs was calculated using the Framingham Stroke Risk Score (FSRS) (Table 1). Higher FSRS indicates greater vascular burden. MBI+ status was contingent on meeting the MBI symptom persistence criterion from consecutive Neuropsychiatric Inventory Questionnaire administrations. Accelerated failure time (AFT) models assessed associations between FSRS and time: 1) to MBI; and 2) from MBI to dementia, adjusted for race, education, marital status, and cognition using propensity score weighting to account for potential confounders. An FSRS*baseline cognitive status (normal cognition[NC]/mild cognitive impairment[MCI]) interaction term determined if FSRS association with incident dementia differed by cognitive status. Subset analyses were conducted for MBI+ participants with NC or MCI at baseline. Time ratios (TRs), expressed as percentages, quantified the effect, with TR values <100% indicating faster progression.

**Result:**

The sample comprised 8519 MBI‐free participants (mean age:73.7±9.0 years; 57.5%female) at baseline. Over a median follow‐up of 3.33 years (range: 0.45 to 8.95 years), 17% of participants developed MBI. Higher FSRS was associated with faster progression to MBI (TR%:97.6, 95%CI:0.97–0.98, *p* < .001), particularly for the domains of decreased motivation (TR:97.6, 95%CI:0.97–0.99, *p* <0.001), affective dysregulation (TR%:98.7, 95%CI: 0.98–0.99, *p* <0.001), and impulse dyscontrol (TR%:99.2, 95%CI:0.99–0.99, *p* = 0.02) (Table 2). The interaction between FSRS and cognitive status suggested a difference in dementia progression amongst MBI+ individuals with MCI and NC (*p* = 0.003). Higher FSRS had a greater effect size for faster progression to dementia in MBI+ participants with NC (TR%:95.41,95%CI:0.92‐0.99, *p* = 0.01) than MCI (TR%:99.41,95%CI:0.98‐1.01, *p* = 0.33).

**Conclusion:**

Vascular burden is associated with faster progression to MBI and dementia. Managing VRFs may delay MBI and dementia onset, particularly in cognitively healthy individuals. Future research should explore interventions targeting VRFs to improve cognitive and behavioral brain health.